# First Detection of Jingmen Tick Virus in Hard Ticks Collected Across Multiple Regions of Italy

**DOI:** 10.3390/v18010006

**Published:** 2025-12-19

**Authors:** Silvia Fabi, Mariachiara Vardeu, Alex Martini, Elisa Franchin, Renata Fagundes-Moreira, Giulia Chiarello, Graziana Da Rold, Federica Gobbo, Federica Obber, Valentina Tagliapietra, Chiara Agostini, Arianna Breda, Elisabetta Valente, Valentina Chisu, Cipriano Foxi, Federica Cavaliere, Rokia Moretti, Annapaola Rizzoli, Ilaria Pascucci, Carlo Vittorio Citterio, Giovanna Masala, Fabrizio Montarsi, Claudia Del Vecchio, Ignazio Castagliuolo, Enrico Lavezzo, Cristiano Salata

**Affiliations:** 1 Department of Molecular Medicine, University of Padova, Via A. Gabelli 63, 35121 Padua, Italy; silvia.fabi@studenti.unipd.it (S.F.); mariachiara.vardeu@studenti.unipd.it (M.V.); alex.martini@studenti.unipd.it (A.M.); elisa.franchin@unipd.it (E.F.); chiara.agostini2900@gmail.com (C.A.); arianna.breda.1@studenti.unipd.it (A.B.); claudia.delvecchio@unipd.it (C.D.V.); ignazio.castagliuolo@unipd.it (I.C.); enrico.lavezzo@unipd.it (E.L.); 2 Istituto Universitario di Studi Superiori, Piazza della Vittoria 15, 27100 Pavia, Italy; 3 PhD National Programme in One Health Approaches to Infectious Diseases and Life Science Research, Department of Public Health, Experimental and Forensic Medicine, University of Pavia, Viale Golgi 19, 27100 Pavia, Italy; renata.fagundes.mm@gmail.com; 4 Microbiology and Virology Diagnostic Unit, Padova University Hospital, Via Giustiniani 2, 35121 Padua, Italy; elisabetta.valente@aopd.veneto.it; 5 Department of Veterinary Medicine, University of Bari, Str. Prov. per Casamassima km 3, 70010 Valenzano, Italy; 6 Laboratory of Medical Entomology and Vector-Borne Diseases, Istituto Zooprofilattico Sperimentale delle Venezie, Viale dell’Università 10, 35020 Legnaro, Italy; gchiarello@izsvenezie.it (G.C.); fgobbo@izsvenezie.it (F.G.); fmontarsi@izsvenezie.it (F.M.); 7 U.O. Ecopathology Wildelife Specialistic Center-SCT2-Belluno, Istituto Zooprofilattico Sperimentale delle Venezie (IZSVe), Via Fiorenzo Tomea, 5, 32100 Belluno, Italy; gdarold@izsvenezie.it (G.D.R.); fobber@izsvenezie.it (F.O.); ccitterio@izsvenezie.it (C.V.C.); 8 Research and Innovation Centre Fondazione Edmund Mach, Via Mach 1, 38098 San Michele all’Adige, Italy; valentina.tagliapietra@fmach.it (V.T.); annapaola.rizzoli@fmach.it (A.R.); 9 Department of Animal Health, Istituto Zooprofilattico Sperimentale della Sardegna, Via Duca degli Abruzzi 8, 07100 Sassari, Italy; valentina.chisu@izs-sardegna.it (V.C.); cipriano.foxi@izs-sardegna.it (C.F.); giovanna.masala@izs-sardegna.it (G.M.); 10 Istituto Zooprofilattico Sperimentale dell’Umbria e delle Marche “T. Rosati”—Sede di Pesaro, Via dei Canonici 140, 61122 Pesaro, Italy; f.cavaliere@izsum.it (F.C.); r.moretti@izsum.it (R.M.); i.pascucci@izsum.it (I.P.)

**Keywords:** Jingmen tick virus, Jingmenvirus, tick, *Flaviviridae*, Italy, epidemiology, real-time PCR, sequencing

## Abstract

Jingmen tick virus (JMTV) is a novel flavi-like virus first identified in 2010 in *Rhipicephalus microplus* in the Jingmen region of Hubei Province, China and has been reported in different Asian countries, Central and South America, Africa, and Europe. Beyond ticks, JMTV has been detected in a range of other arthropods and in vertebrate hosts. In humans, JMTV has been found in patients with Crimean-Congo hemorrhagic fever in Kosovo and Turkey, and in febrile patients with a history of tick bites in China, suggesting it may be a novel human pathogen. To investigate the presence of JMTV in Italy, we developed a One-step real-time RT-PCR assay and applied it to individually screen 1150 ticks collected from northeastern, central, and southern Italy. JMTV RNA was detected in multiple tick species, including *Ixodes ricinus*, *Rhipicephalus bursa*, *Rhipicephalus sanguineus* s.l., *Dermacentor marginatus*, and *Hyalomma marginatum* with a prevalence ranging from 0.52% to 18.42% in questing ticks. The detection of JMTV in ticks from all surveyed areas, indicates that the virus is geographically widespread in Italy. These findings highlight the need for comprehensive surveillance strategies to identify new areas of active virus circulation and to investigate the potential impact of JMTV on public health.

## 1. Introduction

Jingmen tick virus (JMTV) is a novel emerging tick-borne RNA virus, first identified in 2010 in *Rhipicephalus microplus* ticks from the Jingmen region of Hubei Province, China [1]. The International Committee on Taxonomy of Viruses currently categorizes JMTV as an unclassified flavivirus within the family *Flaviviridae*. It is the prototype of the Jingmenvirus group, which includes an increasing number of members detected in various hosts worldwide [1,2,3,4,5,6]. In particular, JMTV shows a wide geographical distribution across Asia, Africa, Europe and the Americas [1,5,6,7].

JMTV particles exhibit a spherical morphology, measuring approximately 70–80 nm in diameter, slightly larger than conventional flavivirus particles (~45 nm). They possess envelopes and distinct surface protrusions. Unlike typical flaviviruses, which contain a single-stranded RNA positive genome, the JMTV’s genome is composed of four segments (i.e., S1, S2, S3 and S4) of single-stranded positive-sense RNA. Segments S1 and S3 encode non-structural proteins NSP1 and NSP2, respectively, sharing high similarity with NSP5 and NSb2-NS3 proteins of *Orthoflavivirus* genus members. S1 contains conserved motifs typical of flavivirus RNA-directed RNA polymerase, S-adenosylmethionine binding, and nucleic acid substrate binding, while S3 features both N-terminal and C-terminal helicase domains as well as an ATP binding site [1,5]. In contrast, segments S2 and S4 encode JMTV-specific structural proteins: S2 encodes glycoprotein VP1, and S4 contains two overlapping open reading frames encoding capsid protein VP2 and membrane protein VP3 [1,5]. Since no known viral sequences exhibit significant similarity with segments S2 and S4 of JMTV, it has been hypothesized that these genomic components may have originated from an extinct progenitor virus or from an as-yet-unidentified viral species [2].

JMTV is primarily transmitted to vertebrate host through the bite of infected tick. In ticks, viral maintenance and dissemination can occur via several routes: by feeding on a viremic host, co-feeding transmission on a non-viremic host, and transovarial transmission to the offspring [2]. Originally identified in *R. microplus*, JMTV has been reported in several tick species, such as *Haemaphysalis longicornis*, *H. campanulata*, *H. flava*, *R. sanguineus* s.l., *R. bursa*, *Hyalomma marginatum*, *Ixodes ricinus*, *I. sinensis*, *I. granulatus*, and *Amblyomma javanense*. Among these, *R. microplus* is the species with the highest reported prevalence of infection [2].

Beyond ticks, JMTV has been detected in a range of other arthropods, such as *Drosophila melanogaster* and mosquitoes (e.g., *Armigeres* spp., *Anopheles* spp., and *Culex* spp.), suggesting a broad arthropod host spectrum and the potential involvement of multiple vectors. Furthermore, JMTV genome has been identified in cattle (e.g., Brazil), bats (e.g., Anatolia), goats (e.g., Kenya), and rodents (e.g., China and USA), and a related variant has been reported in a non-human primate in Uganda. Finally, JMTV RNA has been detected in serum samples from fatal cases of Crimean–Congo hemorrhagic fever (CCHF) in Kosovo and Turkey, and in febrile patients with a history of tick bites in China [2,8,9]. Clinical manifestations range from mild to severe and include acute-onset fever, headache, and malaise. Four JMTV-infected patients were diagnosed positive for JMTV through high-throughput sequencing of skin biopsies, with viral replication in skin tissue confirmed by in situ hybridization. The patients presented with an itchy or painful eschar at the tick-bite site, with or without lymphadenopathy, and histopathology revealed local inflammation with neutrophilic infiltration. Detection of viral RNA in blood samples demonstrated active viral replication and viremia in humans [8]. Additionally, eight cases of JMTV infection were identified based on IgG antibody seroconversion [8], highlighting the need for further investigation into JMTV distribution, pathogenicity, and its potential impact on public health [2,6,8]. Although JMTV seems to be pathogenic for humans, no clinical signs have been reported in JMTV-infected animals [2].

In this study, we report the development of a PCR-based molecular assay targeting the segment 4 of JMTV and its application for screening ticks collected from multiple regions of Italy.

## 2. Materials and Methods

**TaqMan primers and probe design.** All complete genome sequences available for the Jingmen tick virus were retrieved from GenBank and grouped according to their four genomic segments. Multiple sequence alignments were generated for each segment using MAFFT (v7) to identify highly conserved regions suitable for primer and probe design. A conserved region within segment 4 was selected as the target for assay development. Segment 4 was selected due to the absence of homology with other viruses. Primers and TaqMan probe were designed using Primer-BLAST 2.17.0 (NCBI), with parameters optimized for high specificity and minimal secondary structure formation. The TaqMan probe was labeled at the 5′ end with 6-carboxyfluorescein (FAM) as the reporter dye and at the 3′ end with TAMRA as the quencher. All oligonucleotides were synthesized by Biosense s.r.l. (Milano, Italy) (Table 1).

**Preparation and quantification of positive control.** The real-time PCR positive control containing the target sequence selected in the present study was generated by annealing two complementary 82 bp oligonucleotides strands derived from the segment 4 of JMTV (Table 2). The double strand oligonucleotide was cloned in the vector pJET1.2/blunt (Thermo Fisher Scientific, Waltham, MA, USA), following the manufacturer’s instructions, and subsequently amplified in *Escherichia coli* DH5α bacterial cells. The resulting pJET-JMTV plasmid was subject to Sanger sequencing to confirm the presence of the correctness of the insert.

A droplet digital PCR (ddPCR) was employed for the absolute quantification of the pJET-JMTV plasmid using the QX200TM Droplet Digital PCR System (Bio-Rad Laboratories, Berkeley, CA, USA), as previously described [10]. The reaction mixture consisted of 11 µL of ddPCR™ Supermix Bio-Rad, 3 μL of primers-probe mix, 3 μL of Nuclease-Free water, and 5 μL of sample. The primers-probe was prepared by combining: 22.5 μL of forward primer (100 µM), 22.5 μL of reverse primer (100 µM), 5 µL of probe (100 µM), and 250 μL of Nuclease-Free water. Droplets were generated and transferred to a 96-well plate, then subjected to the following cycling: 1 cycle of 10 min at 95 °C, followed by 45 cycles of 30 s at 94 °C and 1 min at 60 °C, and a final 10 min step prior to Droplet reading. Quantification was performed using QuantaSoft^TM^ Software 1.7, which reported the absolute number of copies per microliter (copies/µL) of the initial sample.

**Real-time PCR.** A real-time PCR assay based on TaqMan^TM^ hydrolysis probe was performed using the primers and probe listed in Table 1. The reaction mixture consisted of 12.5 μL of TaqMan™ Universal PCR Master Mix 2X (Thermo Fisher Scientific), 2 μL of primers-probe mix, 10 μL of pJET-JMTV plasmid, and PCR Nuclease-Free water up to the final reaction volume of 25 µL. The primers-probe mix contained: 22.5 μL of forward primer (100 µM), 22.5 μL of reverse primer (100 µM), 5 µL of probe (100 µM) and 250 μL of Nuclease-Free water. Cycling conditions were as follows: 2 min at 50 °C, 10 min at 95 °C (DNA polymerase activation), followed by 40 cycles of 10 s at 95 °C and 1 min at 60 °C. Reactions were run on a QuantStudio™ 7 Pro System (Thermo Fisher Scientific).

**Tick sample collection.** Ticks collected as part of several screening projects on tick-borne microorganisms were included in this study. Specimens were obtained predominantly from the environment, but also from domestic and wild animals, and rarely from humans. Collection sites were located in northeastern (between 2024 to 2025), central (between 2023 to 2025) and southern (between 2024 to 2025) Italy, as well as in Sardinia, one of the two major islands (between 2021 to 2022). Tick morphological identification was performed using taxonomic keys [11,12]. For ticks collected in Sardinia, each specimen was longitudinally incised under aseptic conditions, and one half was homogenized using a mechanical tissue lyser (Qiagen Tissuelyser II, Qiagen, Venlo, The Netherlands)at the “Istituto Zooprofilattico Sperimentale della Sardegna”. All the remaining tick samples were individually homogenized at the University of Padova using the Bead Mill Homogenizer Bead Ruptor EliteTM (OMNI International, Kennesaw, GA, USA). Then, samples were stored at −80 °C.

**Total nucleic acid extraction and One-step real-time reverse-transcriptase (RT)-PCR.** Total nucleic acids were extracted from 200 μL of tick homogenate using the MagNA Pure 96 System (Roche Applied Sciences, Basel, Switzerland), according to the manufacturer’s instructions.

Extracted total nucleic acid were screened for JMTV using a One-step real-time RT-PCR assay with the AgPath-ID™ One-Step RT-PCR Kit (Thermo Fisher Scientific).

Each reaction mixture consisted of 12.5 µL of 2X RT-PCR Buffer, 1 µL of 25X RT-PCR Enzyme Mix, 3 µL of primers-probe mix, 5 µL of extracted nucleic acids, and PCR-grade Nuclease-Free water up to a final volume of 25 µL.

The primers-probe mix contained: 22.5 μL of forward primer (100 µM), 22.5 μL of reverse primer (100 µM), 5 µL of probe (100 µM) and 250 μL of Nuclease-Free water. Cycling conditions were as follows: 10 min at 50 °C for reverse transcription, 10 min at 95 °C for RT inactivation and polymerase activation, followed by 40 cycles of 10 s at 95 °C and 1 min at 60 °C. The pJET-JMTV plasmid was included as a positive control.

## 3. Results

### 3.1. Development of the One-Step Real-Time RT-PCR Assay

Selected primers and probe for the real-time PCR assay are reported in Table 1. The analytical performance of the assay was determined exclusively at the real-time PCR step, using the DNA-based positive control pJET-JMTV, except for specificity testing, which was conducted using the final One-step real-time RT-PCR protocol.

**Figure 1:**
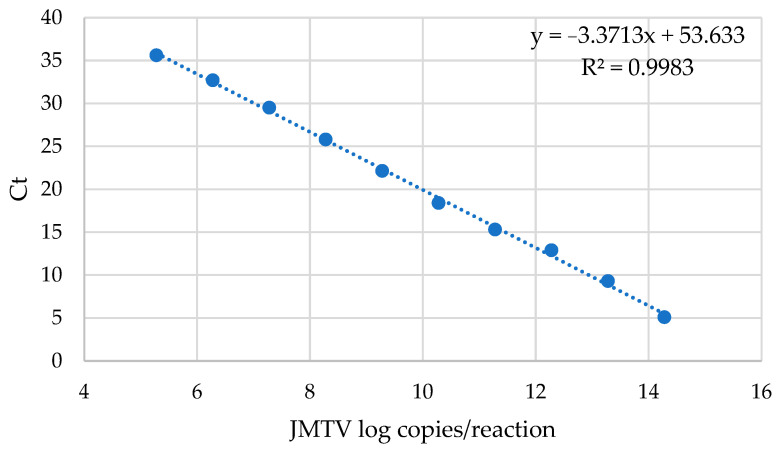
Evaluation on the linearity of the real-time PCR for the detection of JMTV.

To evaluate the linearity of the method, serial tenfold dilutions of the pJET-JMTV plasmid were prepared, and the corresponding threshold cycle (Ct) values were obtained by real-time PCR. A standard curve was generated by plotting the Ct values for each dilution against the Log_10_ of copies/reaction. The assay results show linearity across a plasmid concentration range from 1.92 × 10^14^ to 1.92 × 10^5^ copies/reaction (R^2^ = 0.9983) (Figure 1).

Serial dilutions of the pJET-JMTV plasmid were used to determine the limit of detection (LoD). Plasmid concentration was quantified using droplet digital PCR (ddPCR), yielding 3.83 × 10^14^ copies/µL (15.28 Log_10_ copies/reaction). Subsequently, three dilutions within the linear range were selected and amplified, each in 20 replicate reactions. Results are shown in Table 3. The determination of the LoD value, calculated using StatGraphics 19 software, was determined to be 1. 80 × 10^4^ copies/µL with a 95% probability (lower and upper confidence limits 1.11 × 10^4^ copies/µL and 5.11 × 10^4^ copies/µL, respectively).

To asses assay specificity, the protocol was tested against several flaviviruses, including Dengue virus serotypes 1 and 2, West-Nile virus lineages 1 and 2, Yellow fever virus, Tick-borne encephalitis virus, and Zika virus. Samples used for specificity assessment were quality control materials from the Quality Control for Molecular Diagnostics network (Yellow fever and Zika viruses) or viruses available in the laboratory. The detection of only specific amplifications indicates that the assay demonstrates high specificity.

### 3.2. Tick Identification and Screening for JMTV

#### 3.2.1. Identification of Ticks

A total of 1150 ticks were included in this study. Ticks collected in northeastern Italy were all identified as *Ixodes ricinus* (274 nymphs, 29 females collected in 2024; 72 nymphs and 17 females collected in 2025). Samples from central Italy, collected in 2023–2025 were identified as *I. ricinus* (57 adults and 13 nymphs), *R. sanguineus* s.l. (42 adults), *Dermacentor marginatus* (12 adults), *I. hexagonus* (i.e., eight adults and 11 nymphs), *H. marginatum* (i.e., five adults), *H. punctata* (two adults), *R. bursa* (one adult), *I. canisuga* (one nymph). Ticks collected in southern Italy (2024–2025) were identified as *I. ricinus* (95 females and 55 nymphs) and *H. marginatum* (185 adults). A total of 271 adult ticks were collected in Sardinia. Among these, the most frequently identified species was *R. sanguineus* s.l. (93 individuals) followed by *R. bursa* (66), *H. punctata* (51), *D. marginatus* (25), *H. sulcata* (22), *R. pusillus* (eight), *H. marginatum* (three), *Ixodes* spp. (two) and *I. festai* (one). The sampling locations, tick species, number of tick specimens, and hosts are shown in Supplementary Figure S1.

#### 3.2.2. Tick Samples Testing

**Figure 2:**
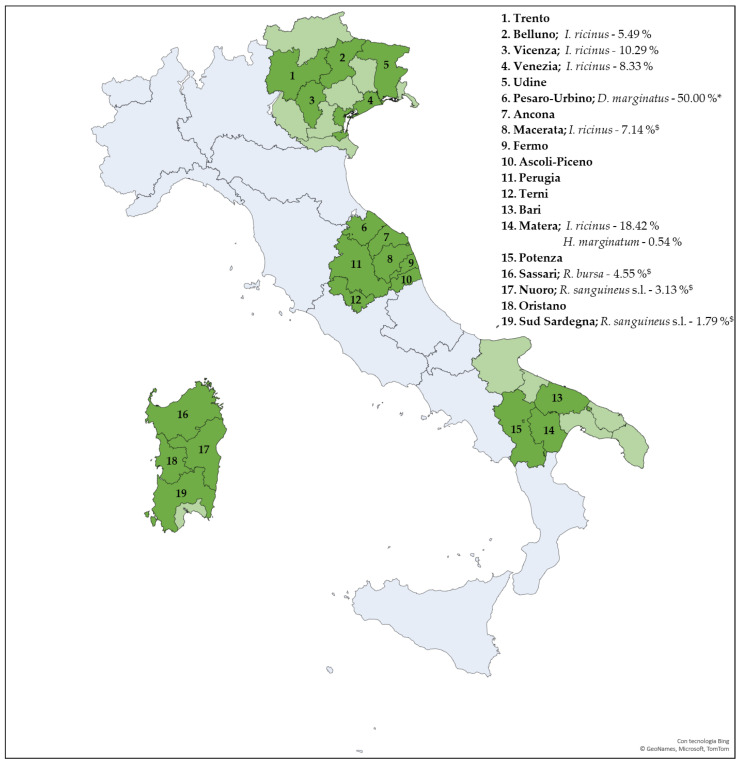
Geographic map of Italy showing the collection locations (Provinces) of tick (dark green), tick species, and the prevalence of JMTV. Light green areas represent Provinces within the administrative Regions where no tick samples were collected. Administrative Regions not included in the study are indicated in light blue. Values marked with an asterisk (*) indicate small sample sizes (2 specimens), while those marked with a dollar sign (^$^) represent data from feeding ticks.

Of the 392 *I. ricinus* specimens collected in northeastern Italy, seven ticks from the municipality of Venice, five from Sedico (Belluno) and 14 from Torrebelvicino (Vicenza) tested positive for JMTV, corresponding to an overall prevalence of 6.63 %. Among the 152 hard ticks collected in central Italy (2023–2025), two *I. ricinus* (Sarnano, Macerata) and one *D. marginatus* (Pesaro-Urbino) were positives (7.14% and 50.00%, respectively). Among the 335 ticks from southern Italy, 14 *I. ricinus* (2025) and one *H. marginatum* (2024) from Basilicata (Parco Regionale Gallipoli Cognato—Piccole Dolomiti Lucane, Accettura, Matera) were positive, corresponding to a prevalence of 18.42% and 0.54%, respectively. Out of the 271 hard ticks collected in Sardinia, two *R. sanguineus* s.l. (Nuoro and Sud Sardegna) and two *R. bursa* (Sassari) tested positive for the virus, resulting in a prevalence of 2.27% and 4.55%, respectively. Administrative Regions included in the study and the prevalence of JMTV-positive ticks for each Province are illustrated in Figure 2 while details on JMTV-positive ticks are reported in Table 4.

To further support the result, amplicons of some positive samples were sequenced to confirm the presence of JMTV (data not shown).

## 4. Discussion

Herein, we report the first detection of JMTV in ticks collected from different areas of Italy using a PCR-based molecular assay targeting the segment 4 of the viral genome. Since its initial discovery in China in 2010, JMTV has been identified in an expanding number of countries worldwide, including Europe regions, in ticks as well as in a variety of arthropod and vertebrate hosts [1,2]. The recent detection of JMTV in Corsica, a French island geographically close to Sardinia, highlights the widespread distribution of the virus and support the likelihood of its presence in Italy. This hypothesis is further reinforced by the detection of JMTV in mosquitoes reared under laboratory conditions in Italy [2,13].

The identification of JMTV RNA in the serum of patients with CCHF and the evidence of viral replication in cutaneous tissues at tick bite sites suggest a potential pathogenic role that warrants further investigation [2,8,9].

The early symptoms described in JMTV-infected patients may easily overlap with those observed in patients affected by tick-borne encephalitis (TBE), which is currently the only clinically relevant tick-borne viral infection recognized in Italy. Therefore, demonstrating the presence of JMTV in areas where TBE is endemic is crucial, as it may introduce additional diagnostic challenge for healthcare providers [9]. Moreover, the development of specific assays for JMTV detection is essential to ensure accurate differential diagnosis in symptomatic patients with a history of tick-bites.

The performance evaluation of our PCR-based molecular assay revealed an LoD of 1.78 × 10^4^ copies/µL, which may result in failure to detect samples with low viral loads. This relatively low sensitivity, compared with a recently published generic RT-qPCR system for JMV detection [13], can be attributed to the need to introduce degenerate positions in both one primer and the probe. This modification was required to address the high nucleotide variability observed among JMTV sequences while preserving the ability to differentiate JMTV from other members of the JMV group. However, LoD values in a similar range have also been reported for real-time RT-PCR systems designed for the specific detection of other JMV group viruses that present high nucleotide variability comparable to JMTV [13].

Our assay demonstrated high specificity, yielding negative results when tested against several flaviviruses, including Dengue virus serotypes 1 and 2, West-Nile virus lineages 1 and 2, Yellow fever virus, Tick-borne encephalitis virus, and Zika virus.

Despite the challenges associated with the LoD, we proceeded with the screening of ticks to assess the presence of JMTV in Italy. To maximize the chances of identifying positive samples, we adopted the strategy of analyzing both adult ticks and nymphs individually.

The detection of JMTV in ticks collected from geographically diverse areas of Italy suggests that the virus is widely distributed across the country. *Ixodes ricinus* nymphs from northeastern and southern Italy presented similar prevalence rates (6.63 % and 4.48%, respectively). In central Italy, the only two positive *I. ricinus* from this area were adult females removed from two roe-deer (*Capreolus capreolus*). Additionally, one questing *D. marginatus* collected in the field was also found to be JMTV-positive, representing the first report of JMTV detection in this species.

In Sardinia, diverse tick species were collected, but only two *R. bursa* and two *R. sanguineus* s.l. tested positive for JMTV. The *R. bursa* specimens were removed from cattle, while *R. sanguineus* s.l. were collected from a sheep and a horse.

Interestingly, the Ct value of the JMTV in *I. ricinus* from southern Italy is better than the Ct values of JMTV of Northern specimens. Several factors could potentially contribute to this variation. These might include, but are not limited to, differences in viral load, variations in sample handling or storage conditions, or potential regional differences in tick populations or virus strains.

Across Europe, JMTV has been detected in *I. ricinus* in continental France [14] as well as in several tick species collected from animals in Corsica [12]. Although our findings provide important evidence of JMTV circulation in multiple Italian regions, further investigation is required to better characterize its distribution and genomic variability across different areas and over time. Moreover, in contrast to *I. ricinus*, the number of individuals belonging to other tick species was limited, and more extensive sampling is required to evaluate the virus circulation in these species.

Even though the detection of JMTV in multiple tick species may indicate that the virus circulates and is maintained in a variety of vectors and hosts, data from feeding ticks cannot provide evidence for the vector role of the tick species, since infection can be acquired previously or during feeding. Similarly, vertebrate host exposure to infected ticks does not provide information on the susceptibility of the host to JMTV infection or on its ability to transmit the virus to other ticks.

In conclusion, we report for the first time the detection of JMTV RNA in ticks collected in different areas of Italy. This finding highlights the expanding geographical range of the virus and emphasizes the need for comprehensive surveillance programs. The widespread presence of JMTV raises significant concerns regarding its potential impact on public health and the need for further investigation into its pathogenicity, transmission dynamics, and potential zoonotic implications. Indeed, future studies will also focus on developing more sensitive molecular assays to improve the detection of JMTV not only in ticks but also in vertebrate hosts, including humans.

## Figures and Tables

**Table 1:** Primers and TaqMan probe sequences employed in the study. The positions of the primers and probe are relative to the GenBank reference sequence OQ320761.

Name	Sequence (5′–3′)	Length (bp)	Product Length (bp)
Primer Fw_899–914_	GGGGACACGCCCAACC	16	70
Primer Rev_968–949_	GGRATCCAACCTTCYCTTCC	20	
Probe_928–948_	TCCCGGAAGACAACACCYACG	21	

**Table 2:** Oligonucleotide sequences utilized to produce the positive control.

	Sequence (5′–3′)
JMTV positive control first strand	TAGAGGGGGGACACGCCCAACCGGTGCCCCCCCGTTCCCGGAAGACAACACCCACGGGAAGGGAAGGTTGGATCCCTGAATG
JMTV positive control second strand	CATTCAGGGATCCAACCTTCCCTTCCCGTGGGTGTTGTCTTCCGGGA-ACGGGGGGGCACCGGTTGGGCGTGTCCCCCCTCTA

**Table 3:** Analytical LoD of JMTV real-time PCR assay using quantified plasmid control.

Dilution	Copy/µL	Copy/Reaction	Ct	N° of Positive Samples
10^−9^	3.83 × 10^5^	1.92 × 10^6^	31.72	20/20 (100%)
10^−10^	3.83 × 10^4^	1.92 × 10^5^	34.96	20/20 (100%)
10^−11^	3.83 × 10^3^	1.92 × 10^4^	37.99	10/20 (50%)

**Table 4:** Information on ticks included in the study: geographic area of sampling, tick species, number of specimens, host/collection method in the field, prevalence (%), and average Ct values. Hosts yielding positive ticks are highlighted in bold. Some prevalence values should be interpreted with caution: values marked with an asterisk (*) indicate small sample sizes, while those marked with a dollar sign (^$^) represent data from feeding ticks.

Areas of Italy	Location (Province)	Tick Species	N° Specimen	Host/Field	Prevalence (%)	Avarage Ct Value
Northeastern IT						
Trentino-Alto Adige	Trento	*Ixodes ricinus*	73	field	0.00	-
Veneto	Belluno	*Ixodes ricinus*	91	field	5.49	34.61
	Venice	*Ixodes ricinus*	84	field	8.33	34.64
	Vicenza	*Ixodes ricinus*	136	field	10.29	34.40
Friuli-Venezia Giulia	Udine	*Ixodes ricinus*	8	field	0.00	-
Central IT						
Marche	Ancona	*Ixodes ricinus*	2	canids/wolf; fox	0.00	-
		*Rhipicephalus* *sanguineus* s.l.	2	canids/wolf; fox	0.00	-
	Ascoli-Piceno	*Ixodes hexagonus*	1	unknown	0.00	-
		*Ixodes ricinus*	5	deer/roe deer; insectivores/hedgehog;	0.00	-
		*Rhipicephalus* *sanguineus* s.l.	3	canids/wolf	0.00	-
	Fermo	*Ixodes hexagonus*	8	European hedgehog	0.00	-
		*Ixodes ricinus*	3	deer/roe deer; canids/fox; canids/wolf	0.00	-
		*Rhipicephalus* *sanguineus* s.l.	3	deer/roe deer; canids/wolf; pigs/boar	0.00	-
	Macerata	*Dermacentor marginatus*	11	boar; cattle; pigs/boar	0.00	-
		*Ixodes canisuga*	1	mustelids; badger	0.00	-
		*Ixodes hexagonus*	4	insectivores/hedgehog; mustelids; badger; wolf	0.00	-
		*Ixodes ricinus*	28	boar; **deer**; deer/fallow deer; deer/roe deer; canids/fox; wolf	7.14 ^$^	35.16
		*Rhipicephalus bursa*	1	cattle	0.00	-
		*Rhipicephalus* *sanguineus* s.l.	16	boar; deer/roe deer; canids/fox; pigs/boar	0.00	-
	Pesaro-Urbino	*Dermacentor marginatus*	2	field	50.00 *	35.36
		*Hyalomma* *marginatum*	6	field; human;	0.00	-
		*Ixodes hexagonus*	2	felids/domestic cat; mustelids/badger;	0.00	-
		*Ixodes ricinus*	17	field; birds/magpie; canids/wolf;	0.00	-
		*Rhipicephalus* *sanguineus* s.l.	14	field; canids/wolf; deer/roe deer;	0.00	-
Umbria	Perugia	*Ixodes hexagonus*	5	European hedgehog; mustelids/badger; rodents/porcupines	0.00	-
		*Ixodes ricinus*	8	deer/roe deer; canids/fox; canids/wolf; wolf;	0.00	-
		*Rhipicephalus sanguineus* s.l.	1	canids/wolf	0.00	-
	Terni	*Haemaphysalis punctata*	2	canids/wolf	0.00	-
		*Ixodes ricinus*	4	canids/wolf; wolf	0.00	-
		*Rhipicephalus* *sanguineus* s.l.	1	canids/wolf	0.00	-
Unknown		*Ixodes ricinus*	2	deer/roe deer; unknown	0.00	-
Southern IT						
Puglia	Bari	*Ixodes ricinus*	49	field	0.00	-
Basilicata	Matera	*Hyalomma* *marginatum*	185	field	0.54	32.29
		*Ixodes ricinus*	76	field	18.42	26.74
	Potenza	*Ixodes ricinus*	25	field	0.00	-
Sardinia	Nuoro	*Dermacentor marginatus*	11	boar; marten, mouflon;	0.00	-
		*Haemaphysalis punctata*	51	goat; mouflon	0.00	-
		*Haemaphysalis sulcata*	22	goat	0.00	-
		*Hyalomma marginatum*	2	boar; human	0.00	-
		*Ixodes spp.*	2	peregrine falcon	0.00	-
		*Rhipicephalus bursa*	10	horse; mouflon	0.00	-
		*Rhipicephalus pusillus*	8	house; fox; marten;	0.00	-
		*Rhipicephalus sanguineus* s.l.	32	boar; dog; fallow deer; **goat**; horse; human;	3.13 ^$^	35.59
	Oristano	*Rhipicephalus bursa*	1	cattle	0.00	-
	Sassari	*Dermacentor marginatus*	13	cattle; human;	0.00	-
		*Ixodes festai*	1	cat	0.00	-
		*Rhipicephalus bursa*	44	cattle	4.55 ^$^	35.15
		*Rhipicephalus sanguineus* s.l.	16	dog; fox	0.00	-
	Sud Sardegna	*Hyalomma marginatum*	1	sheep	0.00	-
		*Rhipicephalus sanguineus* s.l.	56	sheep	1.79 ^$^	34.95
	Unknown	*Dermacentor marginatus*	1	cattle	0.00	-

## Data Availability

The original contributions presented in this study are included in the article. Further inquiries can be directed to the corresponding author.

## Supplementary Materials

The following supporting information can be downloaded at: https://www.mdpi.com/article/10.3390/v18010006/s1. Figure S1. Information on ticks included in the study: geographic area of sampling, tick species, life cycle stage, host/collection in field, number of ticks.
