# Supplementary material for: First Detection of Jingmen Tick Virus in Hard Ticks Collected Across Multiple Regions of Italy
Source: Viruses. 2025 Dec 19;18(1):6. doi: 10.3390/v18010006 (PMC12846579; doi:10.3390/v18010006)
Supplement: Supplementary file 1 [file viruses-18-00006-s001.zip › viruses-4043066-supplementary.pdf]

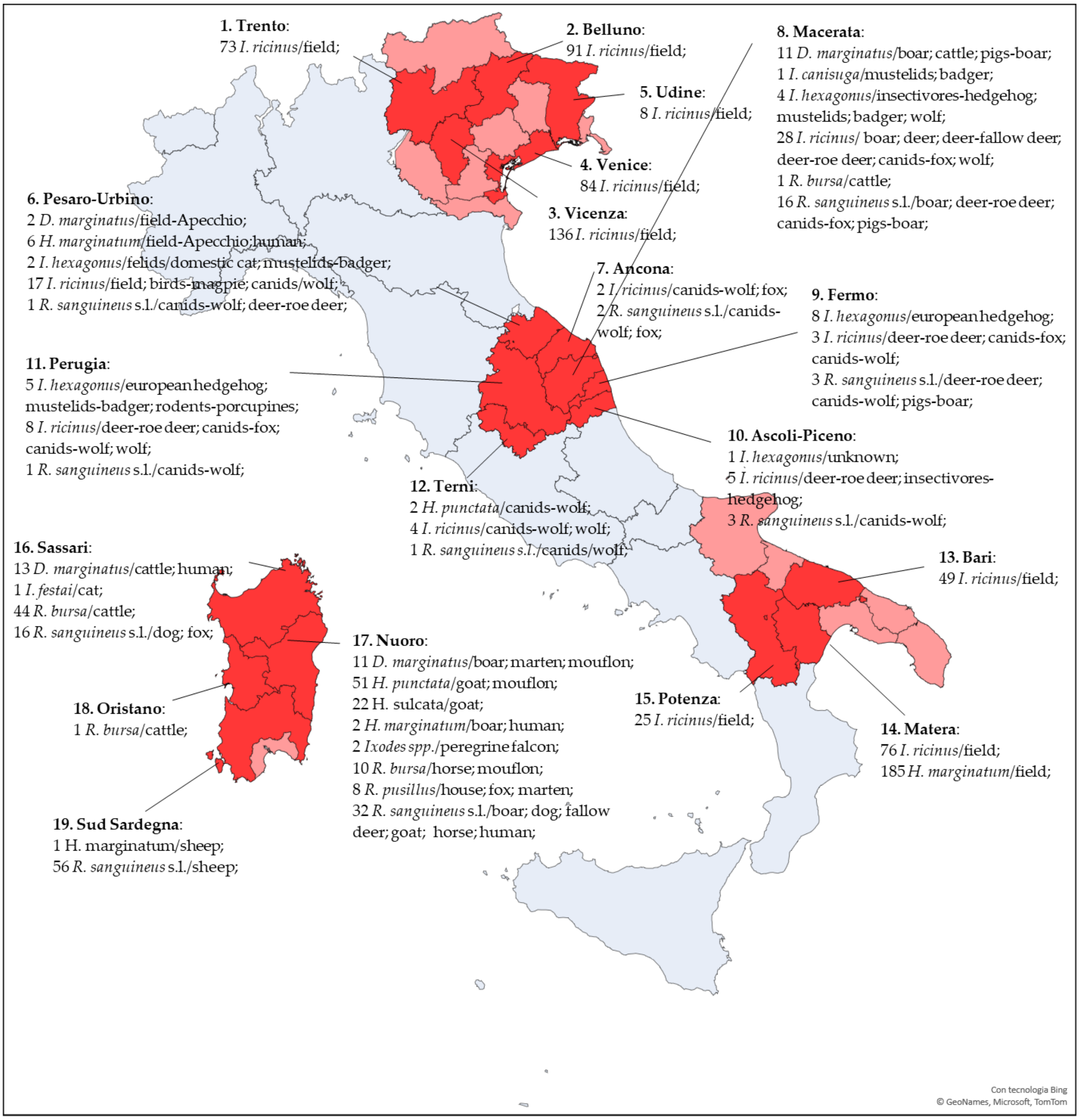

Figure S1: Geographic map of Italy showing sampling locations (Provinces), tick species, number of tick specimens, and hosts.
